# Non-cell autonomous impairment of oligodendrocyte differentiation precedes CNS degeneration in the Zitter rat: Implications of macrophage/microglial activation in the pathogenesis

**DOI:** 10.1186/1471-2202-9-35

**Published:** 2008-04-05

**Authors:** Shin-ichi Sakakibara, Kazuhiko Nakadate, Shigeo Ookawara, Shuichi Ueda

**Affiliations:** 1 Department of Histology and Neurobiology, Dokkyo Medical University School of Medicine, Tochigi, Japan; 2 Department of Anatomy, Jichi Medical University School of Medicine, Tochigi, Japan

## Abstract

**Background:**

The zitter (*zi/zi*) rat, a loss-of-function mutant of the glycosylated transmembrane protein attractin (atrn), exhibits widespread age-dependent spongiform degeneration, hypomyelination, and abnormal metabolism of reactive oxygen species (ROS) in the brain. To date, the mechanisms underlying these phenotypes have remained unclear.

**Results:**

Here, we show differentiation defects in *zi/zi *oligodendrocytes, accompanied by aberrant extension of cell-processes and hypomyelination. Axonal bundles were relatively preserved during postnatal development. With increasing in age, the injured oligodendrocytes in *zi/zi *rats become pathological, as evidenced by the accumulation of iron in their cell bodies. Immunohistochemical analysis revealed that atrn expression was absent from an oligodendrocyte lineage, including A2B5-positive progenitors and CNPase-positive differentiated cells. The number and distribution of Olig2-positive oligodendrocyte progenitors was unchanged in the *zi/zi *brain. Furthermore, an *in vitro *differentiation assay of cultured oligodendrocyte progenitors prepared from *zi/zi *brains revealed their normal competence for proliferation and differentiation into mature oligodendrocytes. Interestingly, we demonstrated the accelerated recruitment of ED1-positive macrophages/microglia to the developing *zi/zi *brain parenchyma prior to the onset of hypomyelination. Semiquantitative RT-PCR analysis revealed a significant up-regulation of CD26 and IL1-β in the *zi/zi *brain during this early postnatal stage.

**Conclusion:**

We demonstrated that the onset of the impairment of oligodendrocyte differentiation occurs in a non-cell autonomous manner in *zi/zi *rats. Hypomyelination of oligodendrocytes was not due to a failure of the intrinsic program of oligodendrocytes, but rather, was caused by extrinsic factors that interrupt oligodendrocyte development. It is likely that macrophage/microglial activation in the *zi/zi *CNS leads to disturbances in oligodendrocyte differentiation *via *deleterious extrinsic factors, such as the cytokine IL1-β or ROS. Atrn might be involved in the activation of brain macrophages/microglia by suppressing excessive migration of monocytes into the CNS, or by accelerating the transformation of brain monocytes into resting microglia. Understanding the pathogenesis of the *zi/zi *rat may provide novel insights into the developmental interaction betweens macrophages/microglia and cells of an oligodendrocyte lineage.

## Background

Emerging evidence suggests that glial cells play an important role in the onset of several neurodegenerative disorders, including multiple system atrophy (MSA), Alzheimer dementia (AD), Parkinson's dementia (PD) and multiple sclerosis (MS). These diseases are characterized by iron deposition in oligodendrocytes, reactive oxygen species (ROS)-mediated oxidative stress, and neuroinflammation accompanied by macrophage/microglial activation in the CNS [1-3].

The zitter (*zi/zi*) rat is an autosomal recessive spontaneous mutant, the phenotype of which includes curled body hair, bent whiskers, fine tremor that develops at 3 weeks of age, and flaccid paresis of the hind limb at around 6 months of age [4]. It has been reported that the levels of ROS production and oxidative stress are abnormally elevated within the *zi/zi *brain [5,6]. Our previous studies suggested that there was a prominent degeneration of dopaminergic neurons in the *zi/zi *rat substantia nigra owing to increased oxidative stress with age [7-9], similar to the degeneration observed in human PD. Pathologically, the *zi/zi *rat brain exhibits severe spongiform degeneration and hypomyelination, which is frequently associated with abnormal membranous structures in oligodendrocytes [10]. A previous study demonstrated that the commencement of myelination was not delayed in *zi/zi *rats compared with control rats, and that the fundamental structures of myelin sheaths are normal in the *zi/zi *rat during the postnatal period [11]. In addition, the biochemical components of myelin, such as myelin basic protein (MBP), proteolipid protein (PLP) and myelin-associated glycoprotein (MAG), are normally expressed [11]. Ultrastructural studies have indicated that the hypomyelination of *zi/zi *rats is characterized by a decrease in the density of myelinated fibers as well as aberrant split myelin lamellae in the ventral column of the cervical spinal cord and in the optic nerve [10,11]. Gross pathological changes in these regions, including vacuole formation and degeneration of myelin sheaths into condensed spheroids, appeared from 3 weeks of age in *zi/zi *rats [10,12]. However, curiously, the incidence of these membranous abnormalities was shown to be quite low; they were seen in only about 5% of the oligodendrocytes in random electron microscopic observations [12]. Consistently, our previous electron microscopic study failed to detect any degenerative changes in the oligodendrocytes in the substantia nigra of *zi/zi *rats until postnatal 4 weeks [9].

A positional cloning study revealed that the *zi/zi *mutant phenotype is due to a severe decrease in the level of *attractin *(*atrn*) mRNA expression as a consequence of an 8-bp deletion at the splice donor site of the gene [13]. *Attractin/mahogany *(*atrn*) mRNA encodes a transmembrane protein containing a C-type lectin domain and four EGF-like motifs, suggesting a function in intercellular interactions through its downstream signaling [14]. *Atrn *also possesses a CUB (C1r/C1s/Urinary epidermal growth factor, Bone morphogenetic protein) domain, which is found almost exclusively in extracellular and plasma membrane-associated proteins. Many CUB domain-containing proteins are proteases, although the roles of the CUB domain have been largely unexplored [15]. In the CNS, although previous *in situ *hybridization studies have indicated a ubiquitous distribution of the *atrn *mRNA in the adult rodent brain [16], it remains obscure what cell-types express atrn protein within the brain, and what its physiological function is in the nervous system. Several pathological studies have suggested the possibility that *atrn *is involved in axon-oligodendrocyte interactions or in both the assembly of myelin sheaths and the maintenance of neurons, but the developmental profile of brain oligodendrocytes of the *zi/zi *rat has not been characterized extensively to date. Moreover, these previous studies have raised the question of whether *zi/zi *hypomyelination is caused primarily by a developmental failure of oligodendrocytes themselves, or alternatively, by secondary consequences of an impairment of neuronal differentiation; they also raise the question of why the incidence of abnormalities in oligodendrocyes is so low (see above).

It has been suggested that atrn also has multiple functions outside the nervous system, including roles in monocyte responses, hair pigmentation, energy control and obesity [17-19]. In the immune system, membrane-type atrn was recently shown to possess dipeptidyl peptidase IV (DP IV)/CD26-like ectoenzyme activity, and to be expressed on the surfaces of human peripheral blood monocytes [20], while the *in vitro *study provided compelling evidence that secreted form of atrn in human plasma has no DP IV activity [21]. CD26 is expressed on the surfaces of hemopoietic stem/progenitor cells, and plays a crucial role in their homing/mobilization ability to/from the bone marrow, through the proteolytic cleavage of a local pool of chemokine SDF-1α [22]. Although atrn exhibits no structural similarity to CD26, it is now thought that atrn is a member of a unique DP IV-family designated the DASH (DP IV activity and/or structure homologues) family based on the comparable substrate specificity of atrn to DP IV [23]. Thus, it is intriguing to find a linkage between the oligodendrocyte degeneration in the *zi/zi *CNS and the tightly regulated expression of atrn in the immune system.

In the present study, we describe the developmental profile of oligodendrocytes and the brain pathogenesis in the *zi/zi *rat in detail. Our observations suggest the possibility that the loss of function in atrn might cause the abnormal infiltration of macrophages/microglia into the brain, mediating the oligodendrocyte morbidity in *zi/zi *rats.

## Results

### Impaired development of oligodendrocytes in *zi/zi *mutants

**Figure 1:**
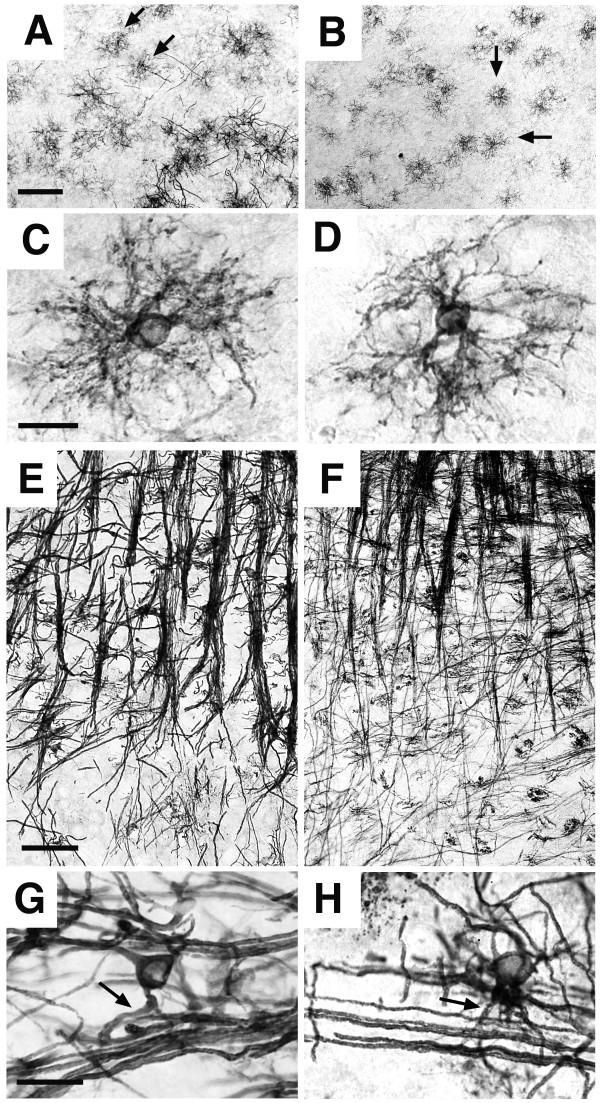
**Myelination in the *zi/zi *hindbrain**. Coronal sections of the pons from *zi/zi *(***B, D, F, H***) or control SD (***A, C, E, G***) rats at P5 were immunostained with antibodies for Rip. ***A, B*, **Medial vestibular nuclei, the developing grey matter in the dorsal region of pons. A large number of Rip-positive promyelinating oligodendrocytes, which had a starburst morphology (*arrows*), were observed in both the control (***A***) and *zi/zi *(***B***). ***C, D***, High-power photomicrographs of each promyelinating oligodendrocytes, respectively. The *zi/zi *promyelinating oligodendrocyte (***D***) exhibited an oval cell body with multiple filopodia and a profuse network of processes, similar to those of wild-type (***C***). ***E, F***, Reticular formation of pons. There were many Rip-positive myelinating oligodendrocytes ensheathing longitudinal axon bundles. ***G, H***, High-power photomicrographs of the myelinating oligodendrocyte in the control (***G***) or *zi/zi *(***H***). Each myelinating oligodendrocyte had the multiple linear processes arrays connected to the cell body by primary processes (*arrows*), indicating that they had commenced axonal ensheathment. Scale bars: 100 μm (A, B); 20 μm (C, D); 100 μm (E, F); 20 μm (G, H).

We first addressed the developmental profile of oligodendrocytes in *zi/zi *rats at postnatal day 5, when the initial myelination is progressing in the CNS, based upon immunohistochemical staining using the antibody Rip, an antigen specific to oligodendrocytes. Rip antibody recognizes the cytoplasm and processes of cells in an oligodendrocyte lineage from their early stages to late differentiated stages; that is, non-myelinating (promyelinating oligodendrocytes), pre-ensheathing immature oligodendrocytes and myelinating oligodendrocytes [24,25]. During the early postnatal development of the CNS, there is a caudo-rostral gradient of increasing oligodendrocyte differentiation and myelination; Rip immunolabelling emerges from the more caudal area to the more rostral area in the CNS. To investigate whether the commencement of myelination is impaired in *zi/zi *brain, coronal sections through the hindbrain regions were immunostained for Rip. In the developing gray matter regions in the pons (vestibular nuclei), there were a large number of promyelinating oligodendrocytes, which is the earliest form of oligodendrocytes recognized by Rip (Fig. 1A,B). These promyelinating oligodendrocytes have a characteristic "starburst" morphology, prior to commencing ensheathment (Fig. 1C,D), as described previously [25]. The number and morphology of promyelinating oligodendrocytes in the *zi/zi *brain appeared to be indistinguishable from those in control rats. At the same time, we observed many Rip-positive myelinating oligodendrocytes extending multiple and linear process arrays ensheathing longitudinal axon bundles within the developing reticular formation of the pons (Fig. 1E,F). Many of these cells often had linear arrays connected to the cell body by primary processes (Fig. 1E,F, *arrows*) and contained both non-ensheathing processes and internodal myelin sheaths, giving them an appearance of cells undergoing myelination. It has been proposed that these myelinating oligodendrocytes subsequently go through a phase of terminal differentiation until the immature internodal myelin sheaths grow symmetrically, and definitive internodal lengths are established [25,26]. Our light microscopic observations revealed that most myelinating oligodendrocytes in *zi/zi *brain show no significant difference in terms of morphology compared with control animals (Fig. 1G,H). These observations indicated that the onset of oligodendrocyte myelination is not adversely affected in the *zi/zi *CNS.

**Figure 2:**
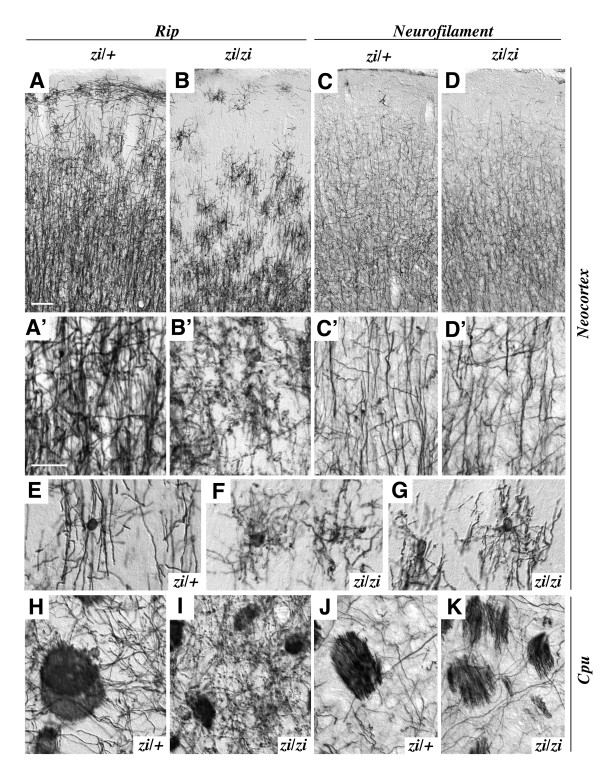
**Developmental defect of oligodendrocytes in the *zi/zi *cerebrum**. Coronal sections of the cerebral cortex (***A-G***) and caudate-putamen (***H-K***) at 4 weeks of age were immunostained with antibodies for Rip (***A, B, E-I***) or neurofilament (***C, D, J, K***). The genotype (*zi/zi *or control *zi/+*) is indicated in each panel. ***A-D***, Cerebral cortex showing Rip-positive oligodendrocytes (***A, B***) and neurofilament-positive axons (***C, D***). The pial surface is at the top. ***A'-D'***, High-power photomicrographs of individual processes of Rip-positive oligodendrocytes (***A', B'***) and neurofilament-positive axons (***C', D'***) in the gray matter (layers II-III). In the *zi/+ *cortex, immunoreactive cells for Rip, which possessed relatively thick and branched processes, were abundantly distributed in cortical layers II-VI, but were sparse in superficial molecular layer I. By contrast, Rip-positive cells in the *zi/zi *cortex were significantly decreased in number throughout the cortex. ***E-G***, High-power views of individual Rip-positive cell in layers I-II. Rip-positive cells *in zi/+ *rats represent the typical morphology of myelinating oligodendrocytes, with round cell bodies and branched processes aligned in parallel (***E***), whereas Rip-positive cells in *zi/zi *rats frequently showed a morphological abnormality of distorted-shaped cell-bodies with fine and wavy processes that branched irregularly (***F, G***). ***H-K***, Caudate-putamen of the basal ganglia immunostained for Rip (***H, I***) and neurofilament (***J, K***). In the *zi/zi *rat, Rip-positive oligodendrocytes exhibited an abnormal morphology with thinner and arborized processes, frequently accompanied by many spherical or granule-like fragments (***I***). Extension of neurofilament-positive axons and axon bundles appeared normal in *zi/zi *rats (***K***). Scale bars: 50 μm (A-D); 20 μm (A'-D', E-K).

**Figure 3:**
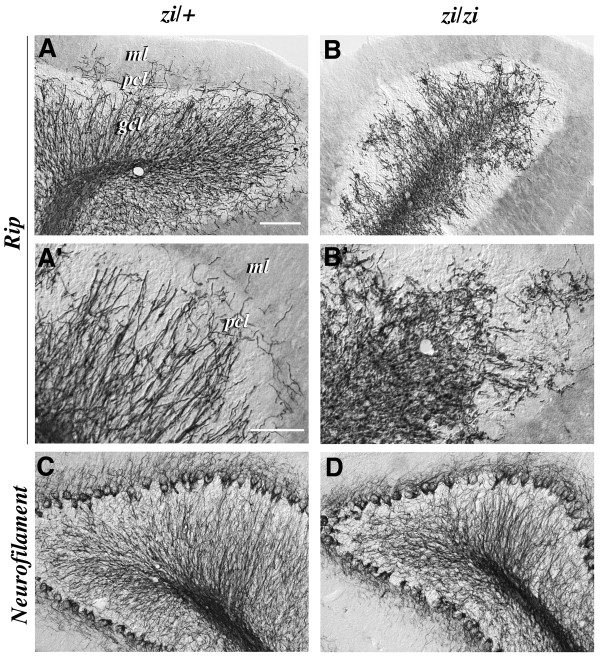
**Developmental defect of oligodendrocytes in the *zi/zi *cerebellum**. Sagittal sections of the cerebellum from *zi/+ *(***A, A', C***) or *zi/zi *(***B, B', D***) rats at 4 weeks of age were immunostained with antibodies for Rip (***A-B'***) or neurofilament (***C, D***). ***A, B***, Differentiating Rip-positive oligodendrocytes were distributed predominantly within the internal granule cell layer (*gcl*) and the white matter tracts in the *zi/+ *cerebellum (***A***), whereas Rip-positive processes of the *zi/zi *mutant cerebellum were distorted and irregularly arranged, and sparsely distributed within the *gcl *(***B***). ***A', B'***, Higher magnifications of the molecular layer (*ml*), Purkinje cell layer (*pcl*) and *gcl *shown in A and B, respectively. The cerebellar oligodendrocytes in *zi/zi *rats exhibited irregularly aligned short processes. ***C, D***, The number, density and distribution pattern of neurofilament-positive axons in *zi/zi *cerebellar folia (***D***) were indistinguishable from those in the *zi/+ *cerebellar folia (***C***). Scale bars: 50 μm (A, B, C, D); 25 μm (A', B'). *gcl*, internal granule cell layer; *ml*, molecular layer; *pcl*, Purkinje cell layer.

We next analyzed the oligodendrocytes in *zi/zi *rats at 4 weeks of age, when the initial phase of myelination had probably just been completed in the forebrain, based upon immunohistochemical staining using antibodies against Rip and neurofilament (NF), a marker for axons. The cerebral cortices of heterozygous control rats (*zi/+*) showed numerous Rip-positive oligodendrocytes throughout the cortex (Fig. 2A). Many Rip-positive oligodendrocytes could be found in the molecular layer of the gray matter and in the white matter of the cerebral cortex. Individual Rip-positive cells had small, round-to-oval cell bodies with characteristic tubular processes branching longitudinally, parallel to the axons, typical of differentiated myelinating oligodendrocytes (Fig. 2A,E). By contrast, the *zi/zi *cortex had a markedly reduced number of Rip-positive cells. In particular, the generation and differentiation of oligodendrocytes was drastically perturbed in the superficial layer, including the molecular layer (layer I) (Fig. 2B). Rip-positive cells that were sparsely distributed in the deeper layers (layers II-VI) of the *zi/zi *rat exhibited severe morphological abnormalities, including distorted cell bodies with fine and wavy processes that branched irregularly (Fig. 2B', F, G). On the other hand, axonal development appeared to be normal in *zi/zi *rats. The axonal extension and density of NF-positive descending and ascending fibers in layers II-V of the *zi/zi *rat were comparable to those in the control rat cortex (Fig. 2C,D). Similar developmental defects of oligodendrocytes were observed in the basal ganglia and cerebellum. In the caudate-putamen of the *zi/zi *rat, Rip-positive oligodendrocytes showed an abnormal morphology of thinner and arborized processes, frequently accompanied by a large number of spherical or granule-like fragments, which were immunoreactive for Rip (Fig. 2I). The extension of NF-positive axons and the appearance of axon bundles in *zi/zi *rats were similar to those in control rats (Fig. 2K). In the control cerebellum, many differentiating Rip-positive oligodendrocytes appeared within the deeper aspects, the granule cell layer and the putative white matter tracts by postnatal 4 weeks, as myelination progressed. These oligodendrocytes and their tubular processes were aligned radially, coursing from the white matter tracts through the Purkinje cell layer to the molecular layer (Fig. 3A, A'). Such a well-organized arrangement of processes could not be found in the *zi/zi *mutant cerebellum (Fig. 3B). Almost all cerebellar oligodendrocytes of *zi/zi *mutants had irregularly aligned short processes in the granule cell layer (Fig. 3B'), suggesting perturbed differentiation of *zi/zi *cerebellar oligodendrocytes. It was also noticed that there were few differentiating oligodendrocytes within the molecular and Purkinje cell layers in the *zi/zi *cerebellum, in contrast to the appreciable number of Rip-positive processes in the corresponding areas of the control rat (Fig. 3A). We failed to find any differences in the distribution profiles of cerebellar axons between *zi/zi *and *zi/+ *rats, at least by postnatal 4 weeks (Fig. 3C,D), as was also the case in the forebrain. In *zi/zi *rats, NF-expressing fibers radially coursing through the granule cell layer, including afferent climbing, mossy fibers and the efferent fibers from Purkinje cells, appeared normal in number and density.

Thus, the spatial distribution of Rip immunoreactivity strongly supported the idea that the loss of function in *atrn *primarily causes defects in oligodendrocyte differentiation within wide areas of the brain, but does not affect neuronal development *per se*, the process of axonal sprouting or axonal projection. Given that the initial phase of myelination is not severely perturbed in the caudal regions of *zi/zi *CNS during the early postnatal period, it is likely that the hypomyelination and aberrant myelin sheath formation in *zi/zi *mutants result from a failure in the terminal differentiation of incipient myelinating oligodendrocytes; that is, defects in the subsequent extension or maintenance of oligodendrocytic processes. Alternatively, it is also possible that the hypomyelination in *zi/zi *brain might be caused by a toxic effect on oligodendrocytes and myelin after its normal formation.

### Iron accumulation in *zi/zi *oligodendrocytes in an age-dependent manner

**Figure 4:**
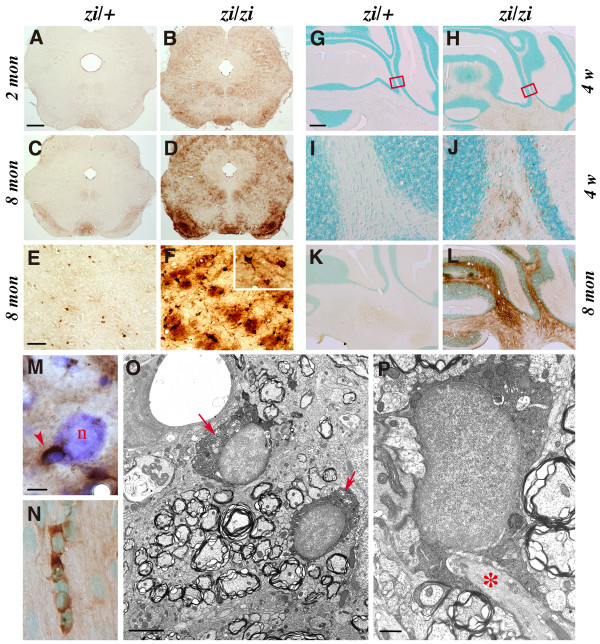
**Iron accumulation in *zi/zi *rat oligodendrocytes**. Accumulating iron was stained using the enhanced Perls' method.**A-D**, Coronal sections of midbrain. Iron was present within the entire midbrain region in *zi/zi *rats (***B***). With increasing age (8 months), iron accumulation became prominent in the substantia nigra (***D***). ***E, F***, Higher magnifications of the substantia nigra region shown in C and D, respectively. In *zi/zi *rats, iron was present in axon bundles and in oligodendrocytic cell bodies and processes (***F***,***inset***). ***G-L***, Iron staining of coronal sections of the cerebellum, counterstained with methyl-green. Iron accumulation was initially detected in the cerebellar white matter at 4 weeks (*4w*) of age (***G, H***). ***I, J***, Higher magnifications of the white matter, shown in the rectangular areas in *G *and *H*, respectively. ***K, L***, Iron deposition increased with age (8 months) within the cerebellar white matter areas in *zi/zi *rats. ***M***, High-power view of iron-containing cells in the *zi/zi *substantia nigra. *Arrow *indicates an iron-accumulating cell that had the morphology of a small and oval cell body with a few short processes, representing an oligodendrocyte. *n *represents a large neuronal cell stained with cresyl-violet. ***N***, High power view of the *zi/zi *cerebellar white matter, showing the cytosolic accumulation of iron (*brown*) in the interfascicular oligodendrocytes. ***O, P***, Electron microscopic photomicrographs of Perls' DAB iron staining of the aged *zi/zi *substantia nigra (8 months), showing iron deposits in cytoplasm of oligodendrocytes (***O, arrows***). The processes of these iron-laden oligodendrocytes frequently encompassed the axon (***P, asterisk***). Scale bars: 1 mm (A-D); 100 μm (E, F, I, J); 500 μm (G, H, K, L); 5 μm (M, N); 1 μm (O); 200 nm (P).

Next, we addressed whether these injured oligodendrocytes produce pathological conditions after brain development in *zi/zi *rats. Previous studies have shown that iron is enriched within oligodendrocytes and myelin under normal physiological conditions [27]. The role that high levels of iron perform within oligodendrocytes has not been fully established, but this elevated iron content has been suggested to promote pathogenesis during disease states such as PD and MS, due to the ability of iron to catalyze the formation of hydroxyl radicals that lead to oxidative tissue damage (for example, lipid-peroxidation and protein oxidation) [28]. Histological examinations have revealed the abnormal accumulation of iron in oligodendrocytes in aged *zi/zi *rats. As shown in Figure 4, the accumulation of iron was observed in axonal elements of white matter tracts, as well as in oligodendrocytes. Prominently affected areas included the substantia nigra (Fig. 4A–F), globus pallidus (not shown) and cerebellum (Fig. 4G–L). Iron accumulation was first observed as early as 4 postnatal weeks within the hindbrain regions of *zi/zi *rats, including the cerebellar white matter (Fig. 4H,J). With increasing age from 2 to 8 months, iron accumulation was accelerated and observed in discrete regions of the *zi/zi *rat white and gray matter. In the substantia nigra pars reticulata, heavy deposition of iron was detected in the cytoplasms of a large number of oligodendrocytes (Fig. 4F*inset*), axon bundles in the vicinity of these cells (Fig. 4F), and peri-neuronal cells (Fig. 4M). In the cerebella at 8 month-old *zi/zi *rats, accumulation of iron was a prominent feature in interfascicular oligodendrocytes (Fig. 4L,N). An electron microscopic study confirmed iron deposits in the cytoplasm and myelin sheaths of oligodendrocytes surrounding axons (Fig. 4O,P).

**Figure 5:**
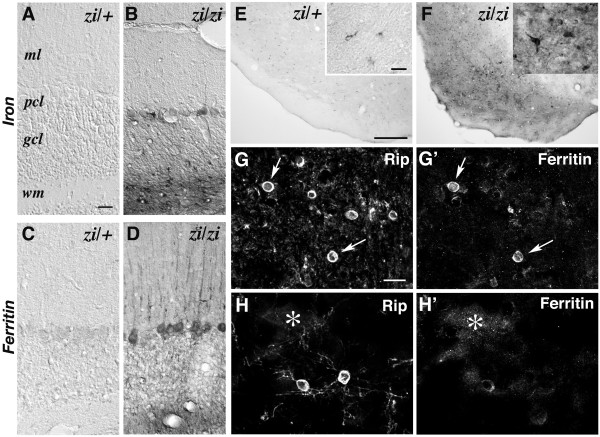
**Up-regulation of ferritin in oligodendrocytes in *zi/zi *rats**. **A-D**, Iron staining (***A, B***) or immunostaining of ferritin (***C, D***) in the cerebella of 8-month-old *zi/zi *(***B, D***) and age-matched *zi/+ *rats (***A, C***). ***E, F***, Immunostaining of ferritin in the substantia nigra of 8-month-old *zi/zi *and *zi/+ *rats. Insets show ferritin-positive cells in the substantia nigra reticulata. ***G-H'***, Confocal images of double labeling for Rip and ferritin in the *zi/zi *substantia nigra, showing the increased expression of ferritin in Rip-positive oligodendrocytes (***arrows***). The diffuse immunoreactivity of ferritin was often observed around the Rip-positive oligodendrocytic processes (***asterisk***). Panels ***G/G' ***and ***H/H' ***represent pairs of double stained photomicrographs. Scale bars: 10 μm (A-D); 500 μm (E, F); 10 μm (insets in E, F); 10 μm (G-H'). *wm*, cerebellar white matter.

To further evaluate the pathologic consequences of the accumulation of excess iron, we compared the pattern of iron staining with that of ferritin expression. Ferritin is a heteropolymeric iron-storage protein composed of H and L subunits that assemble to form a hollow sphere in which ferric iron precipitates are sequestered. In the aged *zi/zi *cerebellum, markedly increased ferritin H/L expression (Fig. 5D) was detected throughout the white matter, and the soma and axons of Purkinje cells where iron accumulated (Fig. 5B). Similar up-regulation of ferritin was observed in small cells located within substantia nigra (Fig. 5E,F). Many of these ferritin-positive cells were Rip-positive oligodendrocytes, as revealed by double immunostaining (Fig. 5G). Notably, scattered and diffused immunoreactivity for ferritin was frequently observed around Rip-positive oligodendrocytic processes, as if there was a leakage of ferritin from these processes (Fig. 5H).

### Absence of attractin expression from cells of an oligodendrocyte lineage

**Figure 6:**
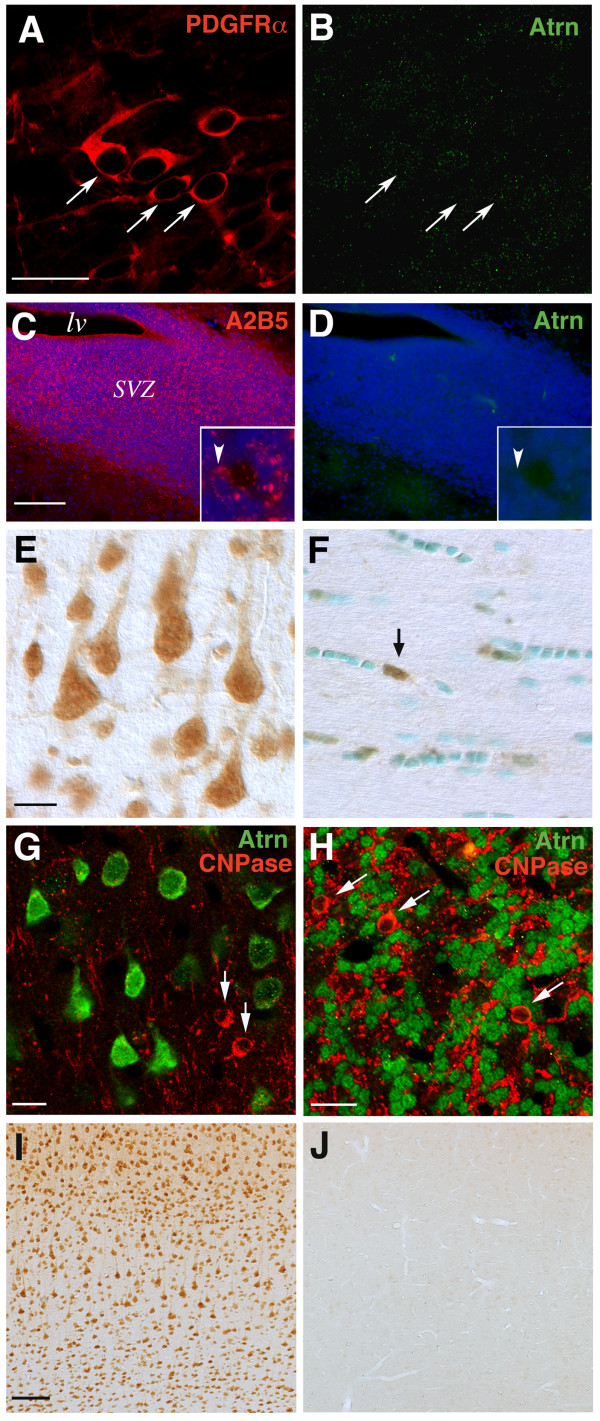
**Absence of atrn protein in oligodendrocyte lineage**. Characterization of atrn-expressing cells in the early postnatal forebrain at P2 (***A***, ***B***), P7 (***C***, ***D***), and in the 2-months-old adult brain (***E-H***). ***A***, ***B***, Confocal images of double immunofluorescence labeling of the subventricular zone (SVZ) surrounding the dorso-lateral corner of the lateral ventricle of wild-type rats, using antibodies against PDGFR-α (*red*) and atrn (*green*). Atrn expression was not detected in PDGFR-α-positive cells (***arrows***). ***C***, ***D***, Confocal images of the wild-type SVZ immunostained for A2B5 (*red*) and atrn (*green*). Cell nuclei were visualized by TOPRO-3 staining (*blue*). *Insets *show an enlarged view of the SVZ. Arrowhead depicts A2B5-positive but atrn-negative OPCs among the densely packed cells in the SVZ. ***E***, Atrn-positive cells (DAB, *brown*) in the gray matter (layer V) of the adult cerebral neocortex. Atrn expression was observed in neurons including typical pyramidal neurons. ***F***, Atrn-positive cell (DAB, *brown*) in the corpus callosum of the adult forebrain was indicated by ***arrow***. ***G***, ***H***, Confocal images of double immunofluorescent labeling with antibodies to atrn (*green*) and CNPase (*red*) in the adult cerebral cortex (layers II-III) (***G***) or the internal granule cell layer of the adult cerebellum (***H***), respectively. Atrn expression was observed in many pyramidal or granule neurons, but not in CNPase-positive oligodendrocytes (***arrows***). ***I***, ***J***, Immunohistochemical analysis for specificity of anti-atrn antibody in rat brain. Coronal sections through the adult cerebral cortex were immunostained with anti-atrn antibody. Atrn-immunoreactivity in wild-type SD (***I***) and *zi/zi *(***J***) rats. Scale bars: 20 μm (A, B); 100 μm (C, D); 10 μm (E, F); 10 μm (F); 20 μm (G); 100 μm (I, J). *lv*, lateral ventricle.

The distribution of atrn protein in the nervous system remains unclear, as there has been no available anti-atrn antibody with which to localize atrn in brain sections, immunohistochemically. In order to examine the precise localization of membrane-bound atrn protein in the CNS, polyclonal antiserum (anti-atrn) was generated in rabbits using a peptide corresponding to the carboxy-terminal 14 amino acids of rat atrn [29]. This antibody recognized an approximately 160 kDa protein, membrane-bound type atrn, but did not cross-react with secreted atrn. Immunohistochemical analysis revealed that atrn was widely, but heterogeneously, distributed. Prominent expression of atrn was found in a large number of neurons, including pyramidal neurons in the neocortex (Fig. 6E) and granule neurons in the cerebellum (Fig. 6H). In most neurons, atrn protein was predominantly distributed in the soma and proximal dendrites. Next, we examined whether atrn is expressed in cells of an oligodendrocyte lineage in wild-type brain. A2B5 antigen and platelet-derived growth factor receptor-α (PDGFRα) are markers of oligodendrocyte progenitors (OPCs) and pro-oligodendrocytes, while galactocerebroside (GC or O1 antigen) and 2', 3'-cyclic nucleotide 3'-phosphodiesterase (CNPase) are used to detect premyelinated and mature oligodendrocytes. PDGFR-α-positive cells residing in the subventricular zone (SVZ) through the first postnatal week have been proposed to be the earliest form of oligodendrocyte progenitor cells that divide in the SVZ to give rise to slightly more differentiated cells of an oligodendrocyte lineage in nascent parenchyma such as the corpus callosum and the cortex [30]. At postnatal day 2 (P2) and 7 (P7), we could not detect any immunoreactivity to anti-atrn within the brain parenchyma, including germinal cells, which were densely packed within the SVZ (Fig. 6A–D).

Double-labeling experiments demonstrated that atrn was not expressed by PDGFR-α- or A2B5-positive cells (Fig. 6A–D), confirming the absence of atrn protein in OPCs at the early postnatal stage. During the process of cellular differentiation, atrn protein is likely to be missing in cells of an oligodendrocyte lineage. Consequently, atrn was found to be absent from almost all differentiated oligodendrocytes in the mature brain (Fig. 6F–H). Double immunofluorescence staining revealed non-overlapping expression of atrn and CNPase. Virtually all CNPase-positive oligodendrocytes in the neocortex (Fig. 6G), striatum (data not shown) and the granule cell layer of cerebellum (Fig. 6H) were devoid of staining for atrn. However, it should be noted that there was a small population of oligodendrocytes that were positive for atrn in the adult brain. For example, atrn was expressed in approximately only 2–5% of the oligodendrocytes within the corpus callosum. The vast majority of these cells, which formed clusters in rows, have small and oval cell bodies and resembled typical myelinating oligodendrocytes, were immunonegative for anti-atrn (Fig. 6F). The specificity of the anti-atrn antibody we used in this report was validated by immunohistochemical analysis using wild-type and *zi/zi *mutant brains. Since we could not find any anti-atrn immunoreactivity in *zi/zi *mutant brain sections (Fig. 6I,J and ref. [29]), the possibility that the cellular heterogeneity of oligodendrocytes (positive or negative for atrn) might result from a staining artifact could be excluded. These observations suggest the possibility that atrn does not exert an intrinsic function in most oligodendrocytes residing in the postnatal brain. The detailed characterization of the anti-atrn antibody, together with an analysis of the spatio-temporal expression pattern and subcellular distribution of atrn, has been described in our resent report [29].

### Normal development of *zi/zi *oligodendrocytes *in vitro*

**Figure 7:**
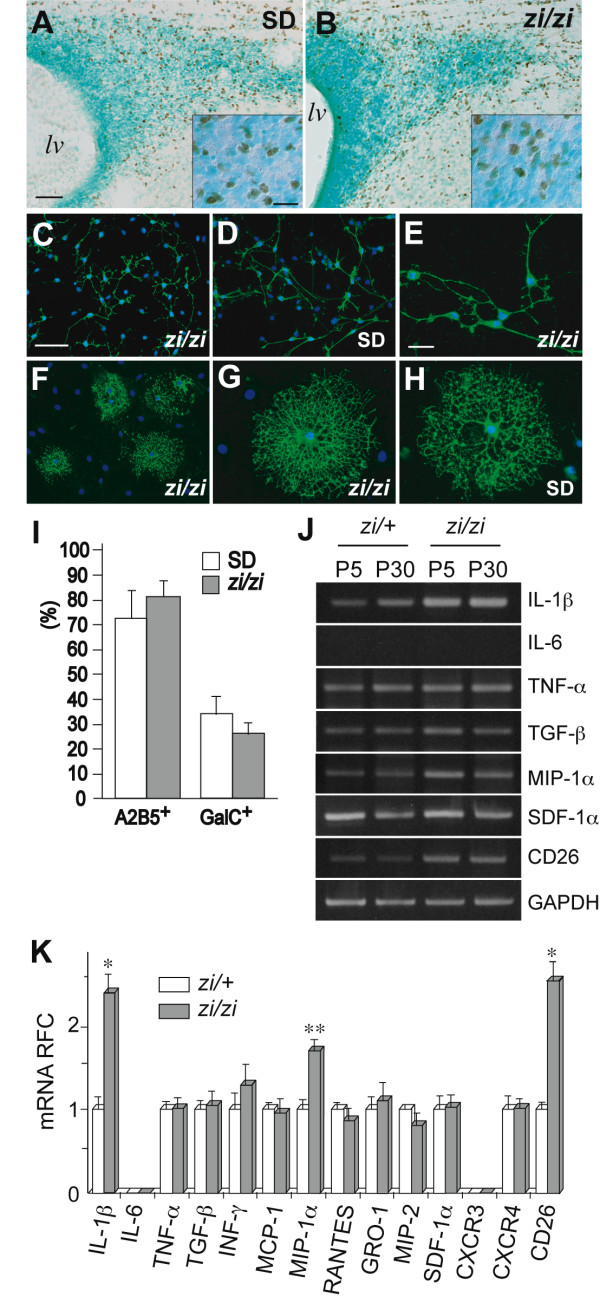
***In vitro *differentiation assays of oligodendrocytes and semiquantitative analysis of cytokine/chemokine mRNAs**. ***A, B***, Distribution of Olig2-positive glial progenitors (*brown*) in the SVZ surrounding the lateral ventricle in the wild-type (SD) rat (***A***) or *zi/zi *rats (***B***) at P7. Lateral is to the right and dorsal is up. *Insets *indicate higher magnifications of Olig2-positive cells lying in the SVZ. ***C-I***, Primary culture of OPCs and subsequent *in vitro *differentiation assays. ***C, D***, OPCs from *zi/zi *(***C***) or control wild-type rat brains (***D***) were immunostained with A2B5 (*green*). Nuclei were counterstained with Hoechst dye (*blue*). ***E***, Higher magnification of A2B5-positive OPCs prepared from *zi/zi *pups, representing a bipolar morphology. ***F***, A differentiated culture of oligodendrocytes from *zi/zi *rats was immunostained with GalC (*green*). ***G, H***, Higher magnification of GalC-positive cells induced from *zi/zi *OPCs (***G***) or control OPCs (***H***). ***I***, Quantification of the fractions of A2B5-positive OPCs and GalC-positive differentiated oligodendrocytes. The ratios of the number of cells positive for each marker to the total number of cells were calculated and presented as the mean ± SEM (%). Scale bars: 100 μm (A, B); 20 μm (inset in A); 50 μm (C-E); 20 μm (F-H). ***J***, Semi-quantitative RT-PCR analysis of cytokine, chemokine and CD26 mRNAs. Total RNA samples prepared from whole brains of *zi/+ *or *zi/zi *rats, at P5 or P30, were subjected to RT-PCR analysis using the primer pairs indicated at the right side of the panels. RT-PCR for *gapdh *mRNA was used as an internal control. A typical electrophoresis pattern of the RT-PCR products is shown on an agarose gel. ***K***, The mRNA levels in P5 brains were expressed as relative fold change (RFC) ± SEM. unpaired *t*-test: **, *P *< 0.05; *, *P *< 0.01; compared with control *zi/+ *rats.

To examine whether the numbers and distribution of glial progenitors, including OPCs, were distorted in *zi/zi *rats *in vivo*, we examined Olig2 expression in the SVZ in the early postnatal forebrain (P7). The basic helix-loop-helix transcription factor Olig2 is specifically expressed in gliogenic progenitors in the early postnatal SVZ [31]. In the *zi/zi *forebrain at P7, Olig2-positive cells were widely dispersed in the SVZ region of the lateral ventricle in addition to the subcortical white matter and developing striatum (Fig. 7). The distribution pattern and number of glial progenitors within the *zi/zi *SVZ expressing Olig2 were indistinguishable from those of the control rat (Fig. 7A,B). This observation allowed us to rule out the possibility that a decreased number of glial progenitors, including OPCs, in *zi/zi *brain contributes to the developmental defects in the oligodendrocyte lineage.

Considering the lack of atrn in cells of an oligodendrocyte lineage, and the normal distribution of Olig2-positive OPCs in the *zi/zi *SVZ, we upheld a hypothesis that the impaired developmental failure of *zi/zi *oligodendrocytes might be caused by a non-cell autonomous mechanism; that is, that some external factor(s) that was increased or decreased in concentration outside the oligodendrocytes in *zi/zi *rats detrimentally influences oligodendrocyte development. To assess this possibility, we next examined the developmental potential and the ability for terminal differentiation of *zi/zi *oligodendrocytes *in vitro*, using a primary culture of purified OPCs. Purified OPCs were cultured from neonatal *zi/zi *brains in serum-free defined medium containing the mitogens PDGF and bFGF; thereafter, these progenitors were induced to differentiate into mature oligodendrocytes. Cultures were used for study only if astrocytes comprised <1% and microglia <0.5% of the total cell number, thus eliminating the effect of other glial cell types. Cultures were characterized immunocytochemically using antibodies against the surface antigens A2B5 and galactocerebroside GalC, sequential expression of which defines early progenitors (OPC) and terminally differentiated oligodendrocytes, respectively, as described previously [32]. As shown in Fig. 7, A2B5-positive OPCs prepared from *zi/zi *rats displayed a typical bipolar or poorly branched morphology (Fig. 7C,E), and could proliferate normally in the presence of mitogens, similar to OPCs from wild-type SD rats (Fig. 7D) (percentage of cells that were A2B5 positive: *zi/zi *rats, 81 ± 6.1% *vs *SD rats, 73 ± 8.6%) (Fig. 7I). In a differentiated culture, there was no difference in the frequency of OPCs differentiating into GalC-positive cells between *zi/zi *and wild-type rats (percentage of cells that were GalC positive: *zi/zi *rats, 26 ± 3.4% *vs *SD rats, 32 ± 5.2%) (Fig. 7I). GalC-positive oligodendrocytes developed from *zi/zi *OPCs exhibited a morphologically mature cell shape and a profuse network of processes and membranes, similar to oligodendrocytes from wild-type rats (Fig. 7F–H). The number of astrocytes or microglia did not increase with differentiation of the cultures (data not shown). These results indicated that cells of an oligodendrocyte lineage from *zi/zi *rats possess normal potential for proliferation and differentiation, at least *in vitro*, and supported the idea that the abnormal development of oligodendrocytes in *zi/zi *rats is not caused by a failure of the intrinsic program in oligodendrocytes themselves, but rather, by changes in extrinsic factors that can influence oligodendrocyte development.

### Changes in the expression of proinflammatory cytokines/chemokines in *zi/zi *brains

One group of candidate extrinsic factors that affect oligodendrocyte development is the proinflammatory cytokines and chemokines. Some proinflammatory cytokines or chemokines, oversupplied from other CNS or non-CNS regions, may exert their toxic effects directly on oligodendrocytes in *zi/zi *rats from an early developmental stage. To determine the levels of various cytokines in *zi/zi *brains, semi-quantitative RT-PCR was performed for interleukin-1β (IL-1β), interleukin-6 (IL-6), interferon-γ (INF-γ), tumor growth factor β (TGF-β) and tumor necrosis factor-α (TNF-α) at postnatal day 5 (P5) and day30 (P30). We also determined the levels of several CC chemokine mRNAs, including those for MCP-1 (CCL2), MIP-1α (CCL3) and RANTES (CCL5), as well as the levels of the CXC chemokine mRNAs for GRO-1 (CXCL1), MIP-2 (CXCL2) and SDF-1α (CXCL12a), and the two chemokine receptors, CXCR3 and CXCR4. As shown in Figure 7J and 7K, IL-1β mRNA expression was significantly higher (2–4 fold) in *zi/zi *brains than in control brains, even at P5 (*zi/+ *vs. *zi/zi*; 0.392 ± 0.217 vs. 1.575 ± 0.321* at P5, and 0.709 ± 0.212 vs. 1.447 ± 0.315* at P30, values are represented as means ± SEM of the relative expression ratio to the level of GAPDH, P* < 0.01 unpaired *t*-test). Such elevated expression of IL-1β mRNA was observed at least up to 5 months of age (data not shown). The levels of TGF-β, TNF-α and INF-γ (data not shown) mRNAs were unchanged between *zi/zi *and control brains. Expression of IL-6 mRNA was undetectable in both groups. Interestingly, gene expression profiling studies of the cerebral cortex and striatum from *zi/zi *rats, using the Affymetrix Microarray system, indicated a significant up-regulation of IL-1β converting enzyme (ICE) mRNA (data not shown). Among the chemokines we examined, MIP-1α (macrophage inflammatory peptide-1α) mRNA expression was detected at higher levels in *zi/zi *rats at P5 (×1.71 ± 0.128, relative fold change compared with control *zi/+ *rats). We could not detect significant changes in the mRNAs for any other chemokines in *zi/zi *rats.

On the other hand, we unexpectedly discovered a marked upregulation of the mRNA for dipeptidyl peptidase IV (DP IV/CD26) in *zi/zi *rats. The CD26 mRNA level was upregulated approximately 2–3 fold in *zi/zi *postnatal brains (P5 and P30), compared with *zi/+ *brains (Fig. 7K). CD26, a 110 kDa cell surface glycoprotein with a large extracellular domain, a transmembrane segment and a cytoplasmic tail, plays an important role in T cell co-stimulation [33].

### Prolonged activation and accelerated recruitment of macrophages/microglia in the *zi/zi *CNS

**Figure 8:**
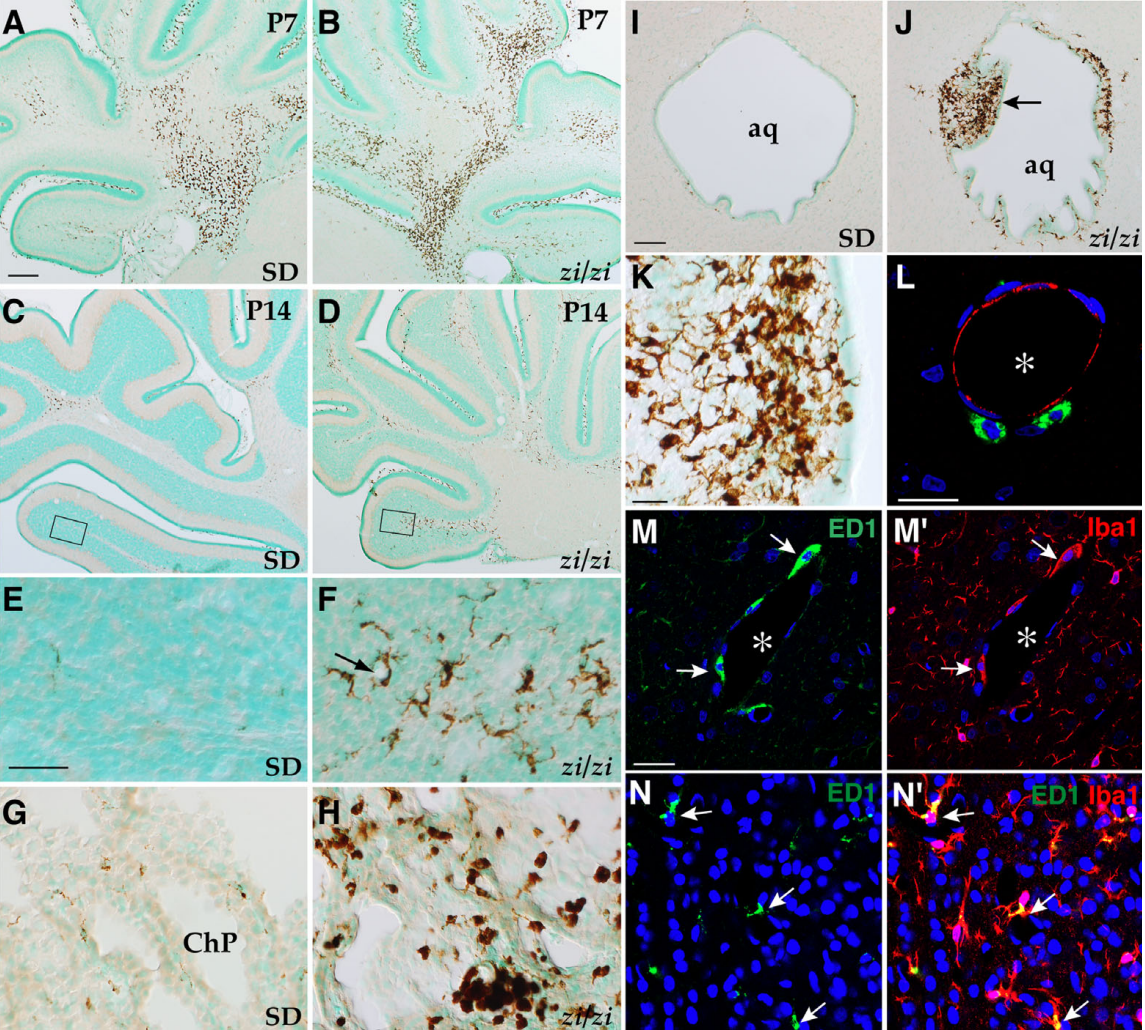
**Accelerated recruitment of macrophages/microglia into the brain parenchyma in *zi/zi *rats during early postnatal period**. ***A-K***, Distribution of ED1-positive cells in wild-type (***A, C, E, G, I***) and *zi/zi *(***B, D, F, H, J-N***) rats. ***A-D***, Coronal sections of the developing cerebellum at P7 (***A, B***) and P14 (***C, D***). ***E, F***, Higher magnification of the boxed regions in ***C ***and ***D***, respectively, showing the ED1-positive cells in the internal granule cell layer of the paraflocculus. ***Arrow ***indicates a capillary. ***G, H***, Choroid plexus in the lateral ventricles at P14. ***I, J***, Coronal sections through the aqueduct of the P14 brain. ***Arrow ***indicates the abnormal accumulation of ED1-positive cells underneath the ependymal layer and the formation of polyp-like protrusions into the aqueductal lumen. ***K***, Higher magnification of ED1-positive cells in the abnormal protrusion shown in ***J***. ***L***, Scanning confocal image of a transverse section of a blood vessel in the *zi/zi *pons at P21, representing the ED1-positive cells (*green*) and vWF-positive endothelial cells (*red*), with nuclear staining by TOPRO3 dye (*blue*). ***Asterisk ***indicates the vascular lumen. ***M, M'***, Confocal images of immunofluorescence labeling with ED1 (*green*) and Iba1 (*red*) across the vasculature in the P21 *zi/zi *cerebellum. ***Arrows ***represent ED1-positive and Iba1-positive elongated cells found in the perivascular region. ***N, N'***, Confocal images of immunostaining for ED1 (*green*) and Iba1 (*red*) through the section of the internal granular cell layer of the P21 *zi/zi *cerebellum. ***Arrows ***indicate the ED1-positive and Iba1-positive ramified microglia. Panels **M/M' **and **N/N' **represent pairs of double-stained photomicrographs. Scale bars: 200 μm (A-D); 50 μm (E-H); 100 μm (I, J); 25 μm (K); 20 μm (L); 20 μm (M, N). ChP, choroid plexus; *aq*, cerebral aqueduct.

MIP-1α is known to be a potent chemoattractant for monocytes and T-cells [34], and the increased expression of several chemokines and cytokines, including MIP-1α and IL-1β has been implicated in the immune cell mobilization seen at the site of CNS injury [35]. In addition, it has been shown that CD26 enhances chemotaxis and transendothelial migration of T cells and monocytes toward CC chemokines [36]. These findings assured us that elevated levels of these mRNAs might be closely associated with an infiltration of immune cells, such as monocytes/macrophages, into the *zi/zi *brain parenchyma. To test this possibility, we examined the dynamics of immune cell populations during the early postnatal stages, prior to the apparent onset of oligodendrocyte defects in the *zi/zi *CNS. The mouse monoclonal antibody OX-6, which recognizes MHC class II antigen, was used to label antigen-presenting cells such as B-lymphocytes, dendritic cells and some macrophages [37]. ED1 antibody recognizes CD68 [38], a phagocytic lysosomal membrane antigen, and is a marker for monocytes, tissue macrophages and activated microglial cells, but not for ramified microglial cells (resting microglia). Although we failed to detect any alteration in the distribution of OX-6-positive cells at P21 (data not shown), we observed a considerable change in the spatio-temporal distribution of ED1-positive cells in *zi/zi *brain parenchyma. At P7, there were a large number of ED1-positive macrophages distributed in the *zi/zi *cerebellum (Fig. 8B) and forebrain, but the number and distribution pattern of these cells appeared to be indistinguishable from those in wild-type control sections (Fig. 8A). In the cerebellum, there was a dramatic increase in the number of ED1-positive brain macrophages observed within the cerebellar fiber tracts, such as the inferior cerebellar peduncle and folia white matter (Fig. 8A,B). These cells were present from birth, with their numbers increasing to a maximum at P7. By P14 in wild-type rat brains, these ED1-positive cells become rapidly decreased in number (Fig. 8C), and thereafter, almost disappeared from parenchyma by the third postnatal week. Such dynamic temporal changes in the distribution pattern of ED1-positive cells appeared to be consistent with the normal distribution of rat brain macrophages described by a previous study [39]. By contrast, it was evident that there was a persistent and prolonged accumulation of large numbers of ED1-positive cells at discrete regions in the *zi/zi *CNS, with most exhibiting a microglia-like morphology (Fig. 8F,H). At P14, when evident oligodendroglial abnormalities still had not appeared in the *zi/zi *pups, at least at the light microscopic level, large numbers of ED1-positive cells were already present in the *zi/zi *CNS, in several fiber tracts including the longitudinal fasciculus in the pons, the spinal trigeminal tracts, the pyramidal tracts in the medulla, and the cerebellar peduncles, in addition to structures outside the blood-brain barrier, including the leptomeninges, choroid plexus and circumventricular organs. Considerable accumulation of ED1-positive cells in these areas was observed, at least beyond the fourth postnatal week in *zi/zi *rats. This distribution pattern of ED1-positive cells appeared to correlate well with the brain areas where many oligodendrocytes show obvious morphological changes. For instance, the development of oligodendrocytes was severely perturbed in the superficial white matter (layer I) of the *zi/zi *cerebral cortex, as shown in Fig. 2. Consistently, we often observed an increased number of ED1-positive cells within layer I, in addition to the leptomeninge located adjacent to layer I (data not shown). However, we failed to demonstrate definitively a direct intercellular association between ED1-positive cells and degenerated oligodendrocytes in *zi/zi *cortex *in situ*, because of the high migrating ability of these invading ED1-positive cells. In the *zi/zi *cerebellar cortex at P14, there were also many scattered EDl-positive cells found widely within the internal granule cell layer, in addition to the folia white matter areas. These cells exhibited an elongated or stellate morphology, extending several ramified cytoplasmic processes, and were often present adjacent to blood vessels (*arrow *in Fig. 8F). At this time, a much lower number of EDl-positive cells could be seen in the cerebellar cortices of control animals (Fig. 8E). The number of ED1-positive cells located in the P14 *zi/zi *cerebellar cortex (paraflocculus) was approximately 12-fold higher than that in controls (control vs. *zi/zi*; 28 ± 13 vs. 358 ± 34*, values are represented as means ± SEM of the number of ED1-positive cells/paraflocculus, P* < 0.01 unpaired *t*-test), while no significant differences were detected between the *zi/zi *and control cerebellar cortices at P7 (control vs. *zi/zi*; 411 ± 57 vs. 483 ± 76). Again, such accumulation of ED1-positive cells in the *zi/zi *cerebellum coincided with the region where oligodendrocyte defects and iron deposition preferentially take place; that is, a large number of ED1-positive cells spread within the internal granule cell layer and the deep white matter tract of *zi/zi *cerebellar folia at P14, prior to the appearance of gross abnormalities of oligodendrocytes (compare Fig. 3B with Fig. 8F) and iron deposition (compare Fig. 4H,J with Fig. 8D,F) in these regions. We also observed many ED1-positive cells in the choroid plexus and in the periventricular regions surrounding the ventricular system in the *zi/zi *CNS. EDl-positive cells in the choroid plexus had large and round cell bodies with few or no cytoplasmic processes (Fig. 8H), giving them an appearance of monocytes/macrophages infiltrating into the cerebrospinal fluid (CSF) from the periphery. In the periventricular regions, it should be noted that large numbers of EDl-positive cells were apposed to and within the ependymal cell layers that surround the cerebral aqueduct at P14 (Fig. 8J). These EDl-positive cells frequently accumulated to form clusters underneath the ependyma, so that distorted large protrusions were invaginated into the aqueductal lumen (Fig. 8J). Individual cells within clusters varied in morphology from those with round cell bodies without processes to those with elongated cell bodies with relatively thick and branched processes (Fig. 8K). Several EDl-positive cells were located within the ependymal cell layers, as if they had just migrated out of the CSF (Fig. 8K). On the other hand, in control rats, EDl-immunoreactive cells were observed surrounding the aqueduct at birth, after which they gradually decreased in number to become almost undetectable by P12, with few ED1-positive cells being detected thereafter (Fig. 8I).

We next attempted to determine the phenotype of EDl-positive cells found in the *zi/zi *parenchyma. Sections across the pons and cerebellum at P21 were processed for immunofluorescence detection with multiple cell type-specific markers, counterstained with the nuclear dye TOPRO3, and analyzed by confocal microscopy. An antibody to von Willebrand Factor (vWF) was used to label the endothelial cells of blood vessels [40], and Iba1 (ionized calcium-binding adapter molecule 1), a calcium binding protein, was used to label resident microglia, including both resting and activated forms [41,42]. As shown in Fig. 8F, a significant number of EDl-positive cells were intimately associated with the vasculature, including capillaries and vessels of larger caliber, throughout the brain. These cells exhibited an amoeboid or elongated morphology, and contained an extensive perinuclear cytoplasm, with few extending projections (Fig. 8L,M). Combined immunostaining with vWF confirmed that these EDl-positive cells resided on the parenchymal side of the endothelium, but never within the lumen (Fig. 8L). Anatomical distribution of these cells suggested that they belong to a subset of macrophages termed perivascular cells, which are not integral components of the vascular wall and are derived from the bone marrow, migrating into the brain parenchyma during postnatal and adult life [43]. Of interest is the observation that almost all ED1-positive perivascular cells were also immunoreactive for the microglial marker Iba1 (Fig. 8M). On the other hand, there were a large number of ED1-positive stellate cells with thick ramified processes in discrete regions of *zi/zi *brain, as shown in Fig. 8F. They were often deeply implanted in the parenchyma, exhibiting no apparent association with vascular elements. Double immunostaining of sections through the *zi/zi *cerebellar gray matter at P21 revealed that these cells were a subpopulation of Iba1-positive microglia (Fig. 8N* arrows*), presumably corresponding to the activated microglial cells characterized, in part, by the ED1-expressing phagocytic lysosomes.

Taken together, recruitment of monocytes/macrophages into the brain parenchyma and the subsequent activation of microglia appeared to be accelerated in the *zi/zi *CNS during the postnatal stage, prior to the appearance of their abnormal clinical signs for hypomyelination.

## Discussion

### Non-cell autonomous impairment of oligodendrocyte differentiation in the *zi/zi *CNS

The present study provides a comprehensive phenotypic profile of the *zi/zi *brain during the early postnatal period. We observed the normal projection of neuronal axons and the developmental failure of oligodendrocyte processes in *zi/zi *rats. Onset of the impairment of oligodendrocyte differentiation might occur in a non-cell autonomous manner in *zi/zi *rats. Aberrant extension of cellular processes and hypomyelination of oligodendrocytes were not due to the failure of the intrinsic program of oligodendrocytes, but rather, were caused by extrinsic factors that interrupt oligodendrocyte development, based on the facts that *zi/zi *OPCs exhibit normal competence for proliferation and differentiation into mature oligodendrocytes in an *in vitro *culture system. The immunohistochemical evidence showing the absence of atrn expression from the early stage of an oligodendrocyte lineage reinforced the idea of non-cell autonomous failure of oligodendrocyte development in *zi/zi *rats. Such a non-cell autonomous defect of oligodendrocyte development may explain why the penetrance of abnormalities in oligodendrocyes is low (*see Background section*). If *zi/zi *oligodendrocytes developed under the control of an impaired intrinsic program, most of these cells should exhibit a common and synchronous abnormality. Nevertheless it should be noted that there was a small population of oligodendrocytes that were positive for atrn in the adult brain [this study, and ref. [29]]. Thus, it is also possible that atrn bears a critical function in some oligodendrocytes. Loss-of-function of atrn in this small population might be sufficient to initiate vacuolation in the *zi/zi *rat. Alternatively, atrn may be transiently expressed in most oligodendrocytes at the time when its function is required. We might have failed to detect atrn expression in oligodendrocytes due to the rapid down-regulation of atrn in this cell lineage. A recent study demonstrated that there is a decline in the amount of plasma membrane lipid rafts in the liver and spleen cells prepared from atrn mutant mice, suggesting a cell-autonomous defect in the plasma membrane maintenance of atrn-deficient cells [44]. Therefore, we cannot exclude the possibility that atrn transiently plays an intrinsic role in the maintenance/integrity of the plasma membrane or the myelin sheaths of oligodendrocytes. Another explanation for the low penetrance of myelin defects observed in *zi/zi *rat is the redundancy of the biochemical pathways in oligodendrocyes in which atrn functions. Atrn and atrnl1 (attractin like-1) gene products were shown to share significant sequence similarity [45]. A genetic study using transgenic and knock-out mice actually demonstrated that over-expression of *atrnl1 *compensates for the loss of *atrn *[45]. Given the similar patterns of expression of the two genes in the adult rodent CNS [45], endogenous atrnl1 might partially compensate to prevent development of a severe phenotype in the *zi/zi *CNS.

We should further note the possibility of inappropriate expression of the secreted form of atrn in the *zi/zi *brain. In the rat brain, the *atrn *gene is transcribed into two different mRNAs with sizes of 9.0 kb and 4.5 kb, by alternative splicing. The 9.0-kb transcript was deduced to encode membrane-type atrn, while the 4.5-kb transcript was deduced to encode the secreted type of atrn corresponding to the secreted form of the human *ATRN *locus product [13,46]. Our current and recent studies have shown the absence of membrane-type atrn protein in *zi/zi *brain using an antibody specific for the cytoplasmic tail of membrane-type atrn [this study and ref. [29]]; however, it remains to be determined whether an abnormal secreted form of atrn protein is generated in *zi/zi *brain. However, we could exclude the possibility that the defect in *zi/zi *oligodendrocytes is due to the inappropriate expression of the secreted form of atrn in the *zi/zi *rat brain for the following three reasons. (i) The deletion in the *atrn*^*zi *^allele has been identified at the splice donor site of the intron of the *atrn *gene, which is expected to result in unstable transcripts. Certainly, previous Northern analysis showed a marked decrease in both secreted-type and membrane-type *atrn *mRNAs in the *zi/zi *brain [13]. Kuramoto *et. al. *also mentioned the existence of faint and long multiple *atrn*-related transcripts in *zi/zi *brain, although it remains unclear whether or not these transcripts encode a functional protein [13]. (ii) Transgenic rescue experiments showed that membrane-type *atrn *is responsible for the neuropathological phenotype in *zi*/*zi *rats, but that the secreted-type *atrn *cannot complement this mutant phenotype [13]. This result indicated that the *zi/zi *brain phenotype is attributable solely to the loss of membrane-type atrn, even though secreted-type atrn might be up-regulated in *zi/zi *brain. (iii) The *mv/mv *(myelin vacuolation) rat, a spontaneous mutant harboring a genomic deletion including exon 1 of the *atrn *gene (*atrn*^*mv*^), showed no detectable expression of both secreted- and membrane-type *atrn *mRNA in the brain [46]. The *mv/mv *rat brain exhibits neurological and neuropathological phenotypes including hypomyelination and vacuolation, which are quite similar to those found in *zi/zi *brain. This finding strongly suggests that the hypomyelination phenotype observed in *atrn *mutant rats is not directly linked to the expression of the secreted form of atrn. Nevertheless, it has been largely unknown whether the secreted form of atrn might exert a specific function in the normal rat and human CNS. Moreover, it is of interest to note that the morphological defects found in the *zi/zi *oligodendrocyte processes appears remarkably similar to the effects of secreted-type human atrn upon dendrite development in differentiating neurons *in vitro *with "wavy processes that branched irregularly" [47]. In order to explore the function of the secreted form in the CNS, an immunostaining study using an anti-atrn antibody recognizing the secreted form is in ongoing in our laboratory.

Our observations raised the possibility that macrophage/microglia activation in the developing *zi/zi *brain leads to the disturbance of oligodendrocyte differentiation in discrete regions of the brain. Primitive macrophages/monocytes have been proposed to be the cells of origin of microglia. These cells are thought to enter the developing brain parenchyma from the bloodstream, the ventricular spaces or the meninges, during both the prenatal and postnatal periods [1,48]. Although the relationship between circulating macrophages and microglial lineage cells is largely undefined, it is possible that the increase in the number of Iba1-positive perivascular cells in the *zi/zi *parenchyma is due to an increase in the population of monocytes/macrophages recruited from the vessels and destined to become microglia. Since both brain macrophages and CNS resident microglia retain common properties, including phagocytic ability, and are possible sources of ROS, proinflammatory cytokines and chemokines, the increased number of monocytes/macrophages/microglia could initially dominate the development of oligodendrocytes within discrete regions of the axon tract through the production of toxic molecules, such as ROS, IL-1β and MIP-1α, leading to the commencement of hypomyelination in the *zi/zi *CNS.

Peroxides, including hydrogen peroxide (H_2_O_2_), are one of the main ROS leading to oxidative stress [49]. A previous biochemical study [5] has shown lowered catalase activity and increased activity of d-amino acid oxidase (d-AAO) in *zi/zi *medulla, pons and cerebellum at P10, before the appearance of morphological vacuolation in the brains of suckling *zi/zi *rats. D-AAO is known to produce ROS such as superoxide anion (O_2_^•-^) and H_2_O_2_, while catalase scavenges H_2_O_2_. These findings suggest the abnormal metabolism of H_2_O_2 _and a subsequent increase in the amount of ROS in *zi/zi *brain. Consistently, our previous studies demonstrated an accumulation of ROS in the nigrostriatal dopaminergic system leading to axonal degeneration or neuronal cell death being frequently observed in the substantia nigra of aged *zi/zi *rats [6,8]. Chronic administration of vitamin E (D, L-α-tocophenol), an effective free radical scavenger in the brain, resulted in a significant increase in the number of surviving dopamine neurons in *zi/zi *brain [6], supporting elevated oxidative stress in *zi/zi *rat brain. Importantly, cells of an oligodendrocyte lineage, especially OPCs, have been shown to be exquisitely vulnerable to oxidative stress [50-52]. Therefore, we assume that free radical injury to developing oligodendrocytes underlies, in part, the pathogenesis of *zi/zi *rats. Presumably, the elevated level of ROS, which are released from accumulated macrophages/microglia, may impede oligodendrocyte differentiation from OPCs during the early postnatal stage, leading to hypomyelination as well as neuronal injury at a later stage.

Proinflammatory cytokines, the mediator molecules of inflammation, such as IFN-γ, TNF-α and IL-1β, have been shown to be derived from macrophages/microglia and have been implicated in the pathogenesis of demyelinating diseases [53,54]. *In vitro *culture studies indicated that the developing oligodendrocytes exhibit higher susceptibility to cytokine-mediated cytotoxicity compared with mature oligodendrocytes [52,55]. IL-1β is a pleiotropic cytokine expressed during normal CNS development and in inflammatory demyelinating diseases. OPCs and differentiated oligodendrocytes are known to express IL-1 receptors, and IL-1β has been shown to block the proliferation of OPCs *in vitro *[56]. As shown here, expression of IL-1β mRNA is elevated from an early stage. This excess delivery of IL-1β may promote the premature differentiation of OPCs in the *zi/zi *brain.

### Possible functions of Atrn in CNS

It is thought that atrn is involved in the initial axon-oligodendrocyte interaction and the assembly process of the myelin sheath, because of the existence of extracellular functional domains, which are usually required for receptor-ligand interactions [14,18]. In this context, however, we cannot explain the possible cellular mechanism by which atrn mediates the activation or invasion of macrophages/microglia in the CNS. It was recently shown that membrane-type atrn possesses an activity of dipeptidyl peptidase IV (DP IV)/CD26-like ectoenzyme, and is expressed on the surface of human peripheral blood monocytes [20], while the *in vitro *study by Friedrich *et al. *provided compelling evidence that atrn protein purified from human plasma has no DP IV activity, suggesting that atrn acts as a receptor or adhesion protein rather than a protease [21]. CD26 is an ectoenzyme DP IV (EC 3.4.14.5) that releases N-terminal dipeptides from peptides with proline in the penultimate position, and is known to be expressed as both a secreted form and a membrane-bound form localized on the surfaces of T cells, B cells and natural killer cells [33]. Several chemokines, including RANTES and SDF-1α, have been identified to be hydrolyzed by CD26 [57]. A recent study suggested that CD26 expressed on the surface of hemopoietic stem/progenitor cells plays a crucial role in their homing/mobilization ability to/from the bone marrow, though the proteolytic cleavage of a local pool of SDF-1α [22]. Although atrn exhibits no structural similarity to CD26, it is now thought that atrn is a member of a unique DP IV-family based on the comparable substrate specificity of atrn to DP IV [20,23]. Our present study indicated that the expression of SDF-1α mRNA in the brain was unaltered in *zi/zi *rats. However, even though atrn cleaves SDF-1α as a target chemokine with the same specificity as CD26, we could not detect such a truncated form of chemokines, as no available technique can discriminate these from the full-length forms. If CD26 serves similar or redundant functions to the *atrn *gene in the CNS, upregulation of its mRNA may reflect a functional compensation for the loss of *atrn *expression in *zi/zi *rats. In this context, the DP IV-activity of atrn may be crucial for the onset or progression of CNS degeneration in *zi/zi *rats. Interestingly, it was shown that atrn potentially influences monocyte function through its DP IV activity; inhibition of DP IV activity in stimulated cultured monocytes caused significantly enhanced release of cytokines [20]. Moreover, inhibition of its activity decreased the adhesion of cultured monocytes to the extracellular matrix [20]. It should also be noted that the secreted form of atrn circulating in human plasma functions in the immune response, mediating the spreading of monocytes that become the focus for the clustering of nonproliferating T lymphocytes [17,58]. Taken together, these findings suggest the possibility that the monocyte/macrophage carrying a mutation of the *atrn *gene may alter the homing/mobilization ability, directing cells towards the chemokines in the brain, or may disregulate their own adhesion properties through the endothelium, leading to extravasation of macrophages into the *zi/zi *brain parenchyma. Alternatively, *atrn *might principally function in promoting the transformation of monocytes into resting microglia in the brain. The accumulation of ED-1-positive monocytes in the *zi/zi *CNS observed in this report might reflect the fact that ED-1-positive cells fail to become resting microglia and remain active in *zi/zi *brain. Thus, there are more ED-1-positive monocytes/macrophages in *zi/zi *rats that express cytokines and chemokines. Considering the expression of atrn in almost all OX42-positive microglia [29], the loss of *atrn *may alter the characteristics of microglia themselves, leading to the continuous and increased production of ROS or cytokines/chemokines in *zi/zi *microglia, and toxicity to oligodendrocytes/myelin as a consequence. Appropriate and persistent expression of atrn in monocytes/macrophages may be required for their proper transform into resting microglia in the brain.

On the other hand, it is well known that the majority of microglia are derived from bone marrow cells during embryogenesis and the early postnatal period. Recent bone-marrow transplantation studies have indicated that there exists slow supplementation of microglia by marrow cells under physiological conditions, even in adult life [43]. Atrn may be involved in brain morphogenesis by suppressing the excessive migration of monocytes into the CNS, or by accelerating the transformation of brain monocytes into resident microglia, but its molecular mechanisms remain to be elucidated. Transplantation studies of EGFP-tagged *zi/zi *macrophages/microglia into the wild-type brain, and co-culture experiments of activated *zi/zi *macrophages/microglia with oligodendrocytes, which are currently in progress in our laboratory, will unequivocally reveal the function of this protein.

### Relevance to neurodegenerative diseases

Iron-mediated oxidative damage to axons may be an early event that is common to several neurodegenerative diseases, such as AD, PD, MSA and MS [3,28]. In the *zi/zi *brain, iron-accumulation became evident in oligodendrocytes with increasing age. Although it is still a matter of debate whether iron accumulation is a primary cause of neurodegeneration or a secondary consequence, there seems to be no doubt that iron-induced oxidative stress contributes to the pathogenesis of neurodegenerative diseases, including animal models [59]. In addition, several lines of evidence also suggest an involvement of macrophages/microglia in these neurodegenerative diseases. Pronounced recruitment and activation of macrophages/microglia into the brain parenchyma have also been implicated in mediating oligodendrocyte and myelin damage, leading to demyelination in MS and its murine model EAE (experimental autoimmune encephalomyelitis) [60]. Recent studies suggest an involvement of CD26/DP IV-activity in the pathogenesis of MS, including T-cell activation, cytokine production and lymphocyte invasion into CNS tissues, through the proteolytic processing of target chemokines [22,33,61]. *In vivo *experiments using a potent synthetic inhibitor of DP IV demonstrated that the clinical signs of EAE could be diminished by DP IV inhibition, both in a preventive and therapeutic fashion, suggesting a crucial role of DP IV activity in the pathogenesis of MS [61]. In view of the compatible DP IV-activity between atrn and CD26, the *zi/zi *rat might be a useful model for studying the functions of DP IV in pathogenesis linking the immune system with nervous system. To date, we have found no hereditary neurological disorders that map onto the human *atrn *gene locus. It will be interesting to determine whether the *atrn *gene is implicated in neurological disorders as a novel modifier.

## Conclusion

Our studies provide evidence that onset of the impairment of oligodendrocyte differentiation occur in a non-cell autonomous manner in *zi/zi *rats. Based on the lack of *atrn *expression from the early stage of an oligodendrocyte lineage, along with the normal competence of *zi/zi *OPCs for proliferation and differentiation *in vitro*, it is highly likely that the hypomyelination of *zi/zi *oligodendrocytes is not due to the failure of the intrinsic program of oligodendrocytes, but rather, is caused by extrinsic factors that interrupt oligodendrocyte development. An enhanced recruitment and prolonged activation of ED-1-positive monocytes/macrophages into the *zi/zi *brain parenchyma during the early postnatal stage, prior to the onset of hypomyelination, suggests the possibility that these activated macrophage/microglial lineage cells might induce disturbances in oligodendrocyte differentiation *via *the secretion of deleterious factors including IL-1β and MIP-1α. Atrn might be involved in the activation of brain macrophages/microglia by suppressing excessive migration of monocytes into the CNS, or by accelerating the transformation of brain monocytes into resting microglia.

## Methods

### Animals

Homozygous zitter mutant (*zi/zi*) rats were maintained as a congenic strain with the genetic background of Sprague-Dawley rats (SD). As control animals, we used age-matched SD rats purchased from Charles River Japan Inc. (Japan), or zitter heterozygote (*zi/*+) littermates, which were obtained by intercrossing each strain. Rats were handled according to the Guidelines for the Care and Use of Laboratory Animals, Dokkyo Medical University School of Medicine.

### Tissue processing

The day of birth was designated as P0. Animals were lethally anaesthetized with sodium pentobarbital administered i. p. (10 mg/kg) and perfused transcardially with phosphate buffered saline (PBS), followed by 4% paraformaldehyde (PFA) in 0.1 M phosphate-buffer, pH 7.4 for immunohistochemistry or by 1% glutalaldehyde, 4% PFA in PB for Perls'/DAB iron staining [62]. Brains and other tissues were dissected, postfixed overnight at 4°C, and cryoprotected in 30% sucrose in PBS at 4°C. Tissue sections for indirect double immunostaining were cut coronally at a thickness of 12 μm using a cryostat, and affixed to 3-aminopropyltriethoxysilane-coated glass slides (Matsunami, Osaka, Japan). For immunostaining with a single primary antibody or Perls'/DAB iron staining, serial free-floating sections were cut at a 30-μm thickness, rinsed with 0.1 M PBS, and stored at 4°C.

### Immunohistochemistry and immunofluorescence

Immunolabeling with a single primary antibody was performed using the avidin-biotin-peroxidase technique (Vectastain ABC kit, Vector Laboratories, Burlingame, CA), according to the manufacturer's instructions. Briefly, free-floating sections were quenched for endogenous peroxidase activity by treating them with 0.3% H_2_O_2 _in PBS for 30 min at room temperature, blocked for 1 h in 10% normal goat serum, and 0.3% Triton X-100 in PBS, and then incubated with a primary antibody diluted in the same blocking solution overnight at 4°C. Sections were placed in an appropriate biotinylated secondary antibody diluted in blocking solution for 2 h followed by incubation with avidin-conjugated horseradish peroxidase (HRP). Immunoreactivities were visualized using 0.25 mg/ml diaminobenzidine (DAB) and 0.03% H_2_O_2_. Each step was followed by four washes in PBS containing 0.3% of Triton X-100 (PBST). For immunostaining with Rip antibody, the sections were quenched with 0.3% H_2_O_2 _after incubation with secondary antibody. Double indirect immunostaining was performed on frozen sections with the appropriate combination of primary antibodies. After four washes with PBST, bound antibodies were visualized by incubation with Alexa Fluor 488- or 568-conjugated secondary antibodies (used at a dilution of 1:1000; Molecular Probes, Inc. Eugene, OR). Cells were counter-stained with 10 μM Hoechst 33342 dye (Sigma-Aldrich, St. Louis, MO) or TOPRO-3 (Molecular Probes) to identify nuclei. After being rinsed with PBST, the specimens were examined under a fluorescence microscope equipped with the appropriate epifluorescent filters. Optical sections were viewed using the FV500 scanning laser confocal imaging system (Olympus, Tokyo, Japan).

### Antibodies

The following antibodies were used in this study: anti-attractin (affinity purified rabbit polyclonal [29]); anti- 2', 3'-cyclic nucleotide-3'-phosphohydrolase (CNPase) (mouse monoclonal IgG_1_, Sigma-Aldrich) diluted 1:200; A2B5 (mouse monoclonal IgM, Chemicon, Temecula, CA), diluted 1:500; O4 (mouse monoclonal IgM, Roche Diagnostics), diluted 1:20; GalC (mouse monoclonal IgG, Sigma), diluted 1:200; anti-PDGFR alpha (rat monoclonal IgG [63]), diluted 1:500; anti-Ferritin H/L (rabbit antiserum, Sigma), diluted 1:1,000; anti-oligodendrocyte-specific protein, Rip (mouse monoclonal IgG_1_, Chemicon), diluted 1:10,000; anti-Neurofilament 70/200 kDa (mouse monoclonal IgG_1_, MP Biomedicals, Costa Mesa, CA), diluted 1:200; ED1 (mouse monoclonal IgG_1_, Chemicon), diluted 1:1,000; anti-Iba1 (rabbit polyclonal antibody, Wako Pure Chemical Industries, Japan), diluted 1:2,000; MRC OX-6 (mouse monoclonal IgG_1_, Serotec, UK), diluted 1:300; anti-vWF (von Willebrand factor; rabbit polyclonal antibody, Dako Japan, Japan), diluted 1:300; and anti-human Olig2 (rabbit IgG, Immuno-Biological Laboratories, Japan), diluted 1:250.

### Oligodendrocyte cultures

Primary rat oligodendrocytes were prepared from the cerebral cortices of *zi/zi *or SD rats at postnatal day 1 using a shaking method [64]. Briefly, forebrains free of meninges were chopped into 1 mm^3 ^blocks and placed into HBSS containing 0.25% trypsin and 10 μg/ml DNase. After digestion for 15 min at 37°C, the tissue was collected by centrifugation and triturated in DMEM medium containing 10% fetal bovine serum, 40 IU/ml penicillin and 40 μg/ml streptomycin, and passed through a 70 μm sieve. Cells were plated onto poly-D-lysine-coated 25 cm^2 ^flasks at a density of 1 pup brain per flask. Cultures were fed with fresh DMEM medium every other day for 10–11 d at 37°C in a humid atmosphere of 5% CO_2_. To isolate oligodendrocytes and oligodendrocyte precursor cells (OPCs), mixed glial cultures were shaken for 1 hr in an orbital shaker at 150 rpm at 37°C to remove adherent microglia/macrophages, and the cultures were washed with the same medium and subjected to continuous shaking at 220 rpm for 16 hr to separate OPCs from the astrocyte layer. The suspension was plated onto uncoated Petri dishes and incubated for 1 hr at 37°C to further remove residual microglia and astrocytes adhering to the dishes. The OPCs were then collected by passing through a nylon mesh, followed by centrifugation. Isolated OPCs were plated onto poly-D-lysine-coated (100 μg/ml) glass coverslips in 24-well plates at a cell density of 2.0 × 10^4 ^cells per well for morphological and immunocytochemical studies. Purified OPCs were cultured for 7–8 d in a serum-free glial defined medium (GDM: DMEM, 0.1% bovine serum albumin (Roche Diagnostics), 50 μg/ml apo-transferrin (Sigma), 50 μg/ml insulin (Invitrogen), 30 nM sodium selenite (Sigma), 10 nM putrescin (Sigma), 10 nM D-biotin (Sigma), 20 nM progesterone (Sigma), 10 nM hydrocortisone (Sigma)), supplemented with 5 ng/ml platelet-derived growth factor (PDGFAA) (R&D systems, Minneapolis, MN) and 10 ng/ml basic fibroblast growth factor (bFGF) (R&D systems). At 7–8 d, the cultures were composed primarily of progenitors and pre-oligodendrocytes (A2B5^+^, O4^-^, GalC^-^). After 7 d, the culture medium was changed to serum-free GDM medium containing 5 ng/ml ciliary neurotrophic factor (CNTF) and 15 nM 3, 3, 5-triiodo-L-thyronine (T_3_) for 7 additional days until cells were differentiated into mature oligodendrocytes. Proliferating OPCs or mature oligodendrocytes were fixed with 4% PFA and immunostained with A2B5 or GalC (O1) antibodies.

### RNA extraction and semi-quantitative RT-PCR assay of cytokine and chemokine mRNAs

At each time point, *zi/zi *or *zi/+ *control rats (n = 3 respectively) were killed and whole brains were rapidly dissected and frozen in liquid nitrogen. Total RNA was extracted using TRIzol reagent (Invitrogen Corp., Carlsbad, CA) according to the manufacturer's instructions and quantified spectrophotometrically. Three micrograms of total RNA was reverse-transcribed according to the manufacturer's protocol (Superscript II, Invitrogen) using random primers, and the cDNA prepared from 30 ng of RNA was subjected to PCR. The primers for rat cytokines were synthesized based on rat cDNA seqeunces for TNF-α (GenBank accession number: 82524821), IL-1β (204905), IL-6 (2170752), TGF-β (11024651), IFN-γ (24475821), CCL5 (RANTES)(124286795), MIP-1α (CCL3) (40254793), MCP-1 (CCL2) (13928713), MIP-2 (CXCL2) (16758459), CXCR4 (82617587), CXCL12 (76496502), GRO-1 (CXCL1) (13540651), CD26 (DPP4) (6978772), CXCR3 (16758151) and GAPDH (9798637) as an internal control. All primer pairs spanned at least one intron in the corresponding genomic DNA. The sequences of oligonucleotide primers and predicted cDNA sizes were as follows: IL-1β, sense 5'-CAAGCACCTTCTTTTCCTTCATC-3' and antisense 5'-GTCGTTGCTTGTCTCTCCTTGTA-3' (241 bp); TNF-α, sense 5'-CCCAGACCCTCACACTCAGAT-3' and antisense 5'-TTGTCCCTTGAAGAGAACCTG-3'(215 bp); INF-γ, sense 5'-GCCAAGGCACACTCATTGAA-3' and antisense 5'-GCTGGTGAATCACTCTGATG-3' (360 bp); IL-6, sense 5'-CAAGAGACTTCCAGCCAGTTGC-3' and antisense 5'-TTGCCGAGTAGACCTCATAGTGACC-3' (101 bp); TGF-β, sense 5'-GAGAGCCCTGGATACCAACTACTG-3'and antisense 5'-GTGTGTCCAGGCTCCAAATGTAG-3' (173 bp); GAPDH, sense 5'-ACCACCATGGAGAAGGCTGG-3' and antisense 5'-CTCAGTGTAGCCCAGGATGC-3' (528 bp); CCL5 (RANTES), sense 5'-CTCCAACCTTGCAGTCGTCT-3' and antisense 5'-GCCTGTGAAGAGCACACCTC-3' (296 bp); MIP-1α (CCL3), sense 5'-TGCTGTTCTTCTCTGCACCA-3' and antisense 5'-GGCTACTTGGCAGCAAACAG-3' (380 bp); MCP-1 (CCL2), sense 5'-TGTAGCATCCACGTGCTGTC-3' and antisense 5'-GCTTGAGGTGGTTGTGGAAA-3' (362 bp); MIP-2 (CXCL2), sense 5'-TCAATGCCTGACGACCCTAC-3' and antisense 5'-ACTCAGACAGCGAGGCACAT-3' (308 bp); CXCR4, sense 5'-GTTTGGTGCTCCGGTAGCTA-3' and antisense 5'-CCAGAAGGGGAGTGTGATGA-3' (317 bp); CXCL12 (SDF-1α), sense 5'-GCTCTGCATCAGTGACGGTA-3' and antisense 5'-CTTTGTGCTGGCAAATCTCAG-3' (382 bp); GRO-1 (CXCL1), sense 5'-AGACAGTGGCAGGGATTCAC-3' and antisense 5'-GAACGACCATCGATGAAACG-3' (375 bp); CD26 (DPP4), sense 5'-GCCTGGGTTTCAGAAGACAGA-3' and antisense 5'-CTGGAACTGGCAGATGTGTTTG-3 (254 bp); and CXCR3, sense 5'-ACAAGTGCCAAAGGCAGAGAAG-3' and antisense 5'-GAGCAGGAAGGTGTCTGTGCT-3' (350 bp). For semiquantitative PCR, target sequences were amplified by 20–35 cycles of PCR (denaturation at 94°C for 30 sec, annealing at 58°C for 30 sec, and extension at 72°C for 30 sec) using a GeneAmp 9700 thermal cycler (Applied Biosystems Japan, Tokyo Japan). The number of amplification cycles was optimized to ensure that PCR products were quantified during the exponential phase of the amplification. A 10 μl aliquot of each PCR product was size-separated by electrophoresis on a 2% agarose gel and stained with ethidium bromide. The gels were photographed, and the bands were quantified using Image J 1.36 software for Macintosh computers and normalized to the values for the housekeeping gene glyceraldehyde-3-phosphate dehydrogenase (GAPDH).

### Enhanced Perls' staining for ferric iron

Iron distribution in *zi/zi *or control brains was examined using the enhanced Perls' method with a minor modification [51]. Free-floating brain sections were incubated in Perl's solution (a 1:1 mixture of 2% potassium ferrocyanide and 2% HCl) for 30 minutes, washed in distilled water, and immersed for 15 min in 0.05% DAB in 0.1 M phosphate buffer (pH 7.4). One ml of 1% hydrogen peroxide was then added for every 200 ml of DAB solution, and sections were incubated in the solution for 15 min. After being rinsed with distilled water for 30 min, sections were mounted on glass slides, lightly counterstained with 0.1% methyl-green or Nissl (0.1% cresyl violet). Some sections were further processed for electron microscopy.

### Electron microscopy

Iron-stained sections were subdissected into smaller rectangular portions that included fields of the substantia nigra within the midbrain. The sections were osmicated with 1% osmium tetroxide solution buffered with 0.05 M phosphate buffer for 2 h and embedded in Epon 812 after dehydration with a graded ethanol series. The specimens were cut using an ultramicrotome and observed using a JEM 2000EX electron microscope (JOEL. Ltd., Japan) after staining with uranyl acetate and lead hydroxide.

## Authors' contributions

SS designed and performed all the experiments, collected and analyzed the data, prepared the figures and contributed to manuscript preparation. KN directed atrn antibody characterization studies. SO conducted the electron microscopic study. SU initiated and headed the project and helped with data interpretation and manuscript preparation.
